# Update of Radiofrequency Ablation for Treating Benign and Malignant Thyroid Nodules. The Future Is Now

**DOI:** 10.3389/fendo.2021.698689

**Published:** 2021-06-24

**Authors:** Ralph P. Tufano, Pia Pace-Asciak, Jonathon O. Russell, Carlos Suárez, Gregory W. Randolph, Fernando López, Ashok R. Shaha, Antti Mäkitie, Juan P. Rodrigo, Luiz Paulo Kowalski, Mark Zafereo, Peter Angelos, Alfio Ferlito

**Affiliations:** ^1^ Division of Otolaryngology – Endocrine Head and Neck Surgery, Johns Hopkins University, Baltimore, MD, United States; ^2^ Department of Otolaryngology – Head and Neck Surgery, University of Toronto, Toronto, ON, Canada; ^3^ Instituto de Investigación Sanitaria del Principado de Asturias and CIBERONC, ISCIII, Oviedo, Spain; ^4^ Division of Otolaryngology - Endocrine Head and Neck Surgery, Harvard University, Massachusetts Eye and Ear Infirmary, Boston, MA, United States; ^5^ Department of Otorhinolaryngology, Head and Neck Surgery, Hospital Universitario Central de Asturias, Oviedo, Spain; ^6^ Institutode Investigación Sanitaria del Principado de Asturias (ISPA), Instituto Universitario de Oncología del Principado de Asturias (IUOPA), University of Oviedo CIBERONC-ISCIII, Oviedo, Spain; ^7^ Head and Neck Surgery, Memorial Sloan-Kettering Cancer Center, New York, NY, United States; ^8^ Department of Otorhinolaryngology – Head and Neck Surgery, University of Helsinki and Helsinki University Hospital, Helsinki, Finland; ^9^ Department of Head and Neck Surgery, University of Sao Paulo Medical School and Department of Head and Neck Surgery and Otorhinolaryngology Department, AC Camargo Cancer Center, Sao Paulo, Brazil; ^10^ Department of Head & Neck Surgery, MD Anderson Cancer Center, Houston, TX, United States; ^11^ Department of Surgery, Bucksbaum Institute for Clinical Excellence, University of Chicago, Chicago, IL, United States; ^12^ The University of Udine School of Medicine, International Head and Neck Scientific Group, Padua, Italy

**Keywords:** autonomously functioning thyroid nodules, benign thyroid nodules, recurrent thyroid cancer, thermal ablation, radiofrequency ablation, primary thyroid cancer

## Abstract

Thermal and chemical ablation are minimally invasive procedures that avoid removal of the thyroid gland and target symptomatic nodules directly. Internationally, Radiofrequency ablation (RFA) is among one of the most widely used thermal ablative techniques, and is gaining traction in North America. Surgery remains the standard of care for most thyroid cancer, and in the right clinical setting, Active Surveillance (AS) can be a reasonable option for low risk disease. Minimally invasive techniques have emerged as an alternative option for patients deemed high risk for surgery, or for those patients who wish to receive a more active treatment approach compared to AS. Herein, we review the literature on the safety and efficacy of RFA for treating benign non-functioning thyroid nodules, autonomously functioning thyroid nodules, primary small low risk thyroid cancer (namely papillary thyroid cancer) as well as recurrent thyroid cancer.

## Introduction

New technologies are available to safely ablate thyroid nodules without removal of the gland itself. Laser Ablation (LA) and Radiofrequency ablation (RFA) are the most widely used thermal ablative treatments for solid nodules. High-Intensity Focused Ultrasound (HIFU) as well as Microwave Ablation (MA) are two other modalities that fall under the umbrella of thermal ablative techniques and are gaining traction as longer-term data emerges (1, 2). Although, chemical ablation *via* percutaneous ethanol injection is cheaper and requires less costly equipment, it is considered the treatment of choice for cystic nodules and tends to be less effective for solid nodules (3–5). Each minimally invasive technique has its subtle differences and nuances, but they all share ease of use, effectiveness and safety.

RFA is an outpatient image-guided thermal ablative procedure that is a potential alternative to surgery for treating symptomatic benign nodules (6–39). This approach eliminates the need for general anesthesia, incision, or removal of the thyroid gland, making it an attractive nonsurgical option. RFA has been offered in certain centers since 2000 for the treatment of primary and metastatic liver, lung, bone and kidney tumors and to ablate aberrant conduction pathways in the heart (40–45). Since the Korean guidelines for the ablation of the thyroid with RFA in 2009 and its revised versions in 2012 and in 2017, various international societies have established their own recommendations (17, 22, 36, 46–48). The American Head and Neck Society has initiated a global collaborative consensus guideline which will be published soon.

With the use of local anesthesia and/or sedation, the RFA probe is introduced into the midline of the anterior neck at the level of the isthmus (called the trans-isthmic approach) and the nodule is targeted using the “moving shot technique” where the operator moves the RFA needle back and forth in the nodule while visualizing the hyperechoic changes in the tissue during ultrasound guidance (18, 45, 48). The heat from the electrode tip causes tissue necrosis and fibrosis by introducing a high-frequency alternating current, which raises tissue temperatures to 60 to 100 degrees Celsius (19). Lidocaine injection can be used prior to ablation to anesthetize the thyroid capsule to hydro-dissect, which provides greater distance from the active RFA needle tip and vital surrounding structures and also provides a heat sink around the nodule to prevent injury to these structures. A solution of 5% dextrose (D5W) may be used to hydro-dissect and to create an aqueous barrier for areas that should remain sensate, such as the trachea, nerves or other vital structures.

Once the tissue is ablated, there are obvious sonographic features available to guide the operator in real-time. These include hyperechoic signals and ‘micro-bubbling’ as well as an increase in the generator impedance as the tissue stiffens, indicating coagulative necrosis (17, 23, 49). The operator approaches the tissue as “subunits” from the deepest to the most superficial portion of the nodule. Care is taken to avoid extending the probe beyond the posterior or lateral thyroid capsule to avoid injury to nearby structures, particularly within the “danger triangle” where the recurrent laryngeal nerve is tethered to the trachea close to the posterior-medial aspect of the thyroid (49). Immediate shrinkage of the nodule is appreciated as well as continued progressive shrinkage over a period of months to years. In benign nodules, the volume of the nodule is expected to typically decrease between 50-90%, which can vary due to both operator and tumor factors (7, 20, 21, 30, 34, 35).

Prior to RFA of a benign nodule, at least two benign biopsy results should be done (either FNA or core biopsy) (17, 22, 36, 46–48). For AFTN, the societies differ slightly in their recommendations, but generally one FNA result can suffice if the ultrasound features are consistent with benign ultrasound results (spongiform, cystic, encapsulated, no evidence of extrathyroidal extension or lymphadenopathy) (17, 22, 36, 46–48). If there are worrisome or suspicious sonographic features, but the biopsy result is benign, the FNA should be repeated. Furthermore, if the volume of the thyroid nodule is not completely delineated on ultrasound or doubt exists regarding the extent of the nodule, a CT scan of the neck can help to determine if and how much retrosternal extension is present and whether the patient is an appropriate candidate for RFA.

## Benign Non-Functioning Thyroid Nodules

Many reports have established the short-term efficacy (6-12 months) and safety of RFA for reducing the volume of benign nonfunctioning nodules (50-85%) (18, 20, 21, 26, 30, 50–52). Long-term data are starting to emerge with recommendations for the number of treatments required to maintain volume reduction of benign nodules. Lim et al. demonstrated up to 93.4% volume reduction rate at 4 years post RFA treatment for benign nonfunctioning nodules as well as improved local symptoms and cosmesis (6). In their series, only 5.6% of the nodules demonstrated regrowth, which tended to be at the peripheral margin (6).

Several papers have shown that RFA has the best reduction rate for smaller nodules (volume < 10 mL), with maintained success for up to 2 years (6, 9, 10, 28–31, 53). Deandra et al. found in their long-term series of 215 patients, that the best response was seen in nodules less than 10 mL, showing an 81% volume reduction rate at 5 years after one RFA procedure (31). Progressive shrinkage over time was noted through the five-year follow-up as well as maintenance of improved compressive symptoms and cosmesis (31). Similarly, in a retrospective multi-institutional trial, Bernardi et al. demonstrated in a series of 216 patients a long-lasting effect of a single RFA treatment with a median volume reduction rate of 77% after 5 years (7). Only 12% underwent a second treatment, and regrowth was noted in 20% of treated patients (7). As opposed to smaller nodules, larger benign nodules tend to require more than one treatment. The variability between the results in the literature can likely be attributed to the heterogeneity of nodule size treated across studies, the energy delivered to nodules, the technical expertise of the operator and the learning curve associated with achieving ablation of the nodule margin (54). These important studies provide a framework for counselling patients regarding the long-term effectiveness of RFA as well as the importance of discussing the potential need for retreatment to maintain the desired effect.

## Autonomously functioning thyroid nodules

Benign autonomously functioning thyroid nodules (AFTN) tend to be more variable than for benign non-functioning nodules likely due to the increased vascularity present in AFTNs and the increased possibility for leaving viable remnant tissue at the margin. The literature demonstrates that achieving euthyroidism post RFA is more consistent when the pretreatment volume of the AFTN is small and more homogeneous ultrasonographically. Cesareo et al. compared the reduction in medium sized nodules (>12 mL, n=14) versus smaller sized nodules (< 12 mL, n=15), and found that euthyroidism was achieved in 86% of small nodules versus 45% in medium size nodules (10). Similarly, Cappelli et al. report a volume reduction rate of 73% with TSH normalization in 94% of 17 patients treated with RFA with nodules of an average 7 mL (11). After one RFA session, Cervelli et al. demonstrated a volume reduction rate of 76.4% +/- 16.9% with a 91% (20/22 patients) TSH normalization at 12-month follow-up in 25 AFTNs that were homogenous in volume and of smaller pretreatment size (55). However, Cesareo et al. found only modest results (57%) of TSH normalization post RFA treatment in a systematic review covering 8 studies on 205 AFTNs (12).

When the volume of a nodule is reduced by > 80%, Cesareo et al. found a greater chance for thyroid function normalization and symptom resolution (10, 15). In a multicenter trial, Sung et al. demonstrated this concept with a significant reduction in nodule volume from a pretreatment mean nodule volume of 18.5 ± 30.1 mL to 4.5 ± 9.8 mL (p <0.001) post RFA treatment in a series of 23 AFTNs (44 patients, 23 with AFTN and 21 with a pretoxic nodule) (13). This significant change in nodule volume and thus vascularity resulted in a significant improvement of triiodothyronine, free thyroxine, and thyrotropin in the final follow up (19.9 ± 12.6 months) (13). TSH levels normalized in 81.8% of the study patients without the development of hypothyroidism post RFA. These findings are congruent with those of Baek et al. in a series of 9 patients (4 AFTN and 5 pretoxic nodules) (14).

Even though several studies support targeting smaller AFTNs to ensure success, the correlation between pretreatment nodule size and treatment outcome of AFTNs is still controversial. A recent meta-analysis highlights the efficacy of RFA treatment on TSH normalization (71.2% of patients) and a volume reduction rate of 69.4% at a mean follow-up of 12.8 months. However, in a subgroup analysis, there was no significant difference in TSH normalization or volume reduction rate (VRR) between small or large nodules when divided into groups of 15mL, 18mL and 20 mL (56). Similarly, Bernardi et al. in their series of 30 patients found no significant correlation between baseline volume and treatment response after RFA (15). Thus, it seems that the rate of reduction of the nodule post RFA plays a more important role in the success of achieving euthyroidism rather than the pretreatment nodule size. However, smaller AFTNs seem to be overall most effectively treated and therefore maybe best for the novice operator to initiate RFA treatment. Due to the variability of results with AFTNs, the international guidelines are more cautious when recommending RFA as curative for AFTNs (17, 22, 36, 46, 47). Accumulating evidence from prospective studies will help in the pretreatment counseling of this patient population.

## Surgery *Versus* Radiofrequency Ablation for Benign Nodules

For symptomatic benign thyroid nodules, an open thyroidectomy is the standard of care. However, surgery may not always be the best choice, particularly for older patients who are not ideal surgical candidates or for patients who do not wish to risk hypothyroidism, scarring, hoarseness or surgical recovery time especially for benign disease. In a meta-analysis comparing thermal ablation with conventional thyroidectomy, thermal ablation was safer and had significantly lower incidence of hoarseness, hypothyroidism and postoperative pain (p<0.05) (57). After thermal ablation, patients had significantly better postoperative cosmetic outcome and shorter hospitalization time compared with conventional thyroidectomy (p<0.05) (57). However, in terms of symptom improvement, both options were equally favorable, with no statistical difference between thermal ablation and conventional thyroidectomy (p=0.58), thus showing RFA as a promising treatment option (57).

Other trials, such as the one by Jin et al. found that thermal ablation is superior to conventional thyroidectomy for benign nodules in terms of patient satisfaction, post-operative quality of life, and shorter hospital stay (58). Although both options were found to have similar complication rates, thermal ablation can take longer to achieve the desired volume reduction than a definitive surgical approach. In a telephone survey of 126 patients treated with RFA and 84 treated with surgery for a single benign thyroid nodule, Bernardi et al. compared patient satisfaction (8). Overall, 94% of patients that underwent surgery were fully satisfied with the outcome and resolution of their nodule related symptoms or hyperthyroidism. For nonfunctioning thyroid nodules, RFA was as effective as surgery in patient satisfaction with resolution of nodule-related symptoms. On the other hand, for AFTN, surgery relieved 95.8% of patient’s hyperthyroid symptoms whereas RFA was effective in 52.9% patients resulting in withdrawal of their antithyroid drugs. In terms of complications, however, RFA was superior to surgery and no cases of hypothyroidism were identified, whereas 37.5% of patients after surgery required thyroid replacement therapy (8).

Other studies have compared the complication rates of surgery and RFA for benign nodules and found a higher rate of complications in the surgical group compared to RFA (9). Che et al. compared complications after open surgery versus RFA and found that 71.5% versus 0% had hypothyroidism, 3% versus 0.5% had recurrent laryngeal nerve palsy, and 3% versus 0% had hypoparathyroidism (9). For the accurate calculation of post-surgical recurrent laryngeal nerve injury rates both the American Academy of Otolaryngology and the American Thyroid Association have recommended laryngeal exam postop given the known disparity between vocal cord paralysis and vocal symptoms (59, 60). When we compare surgery to RFA complications moving forward we will need to be steadfast in ensuring that laryngeal exam is preformed uniformly in both treatment groups.

One of the key benefits of RFA compared with surgery is the reduced chance of causing post-treatment hypothyroidism. Surgery involves removing the gland with the nodule and the normal parenchyma, whereas RFA targets the nodule alone, leaving the normal tissue protected and preserving thyroid function (9). Whether RFA changes the tissue planes, making it more difficult to resect surgically at a later date remains to be determined.

Nevertheless, some limitations of RFA should be mentioned. RFA is not suitable for all types of thyroid nodules, particularly for large benign nodules when patients expect rapid results, substernal nodules, and deeply located nodules. Additionally, there are still some nodules with incomplete response and local regrowth in the follow-up period, which require repeat ablation or surgery, and some nodules which shrink slowly but fail to completely recede (61).

## Combined Thermal Ablation and Radioactive Iodine Treatment

The guidelines are more cautious when recommending treatment of large benign goiters, and more than one treatment is expected for larger sized nodules (17, 22, 36, 46, 47). The use of one treatment modality exclusively may only partially give a desired effect for benign large toxic nodules, however evidence for combining thermal ablation with radioactive iodine (RAI) has been promising. Even though surgery is considered the first line treatment, and current international guidelines do not endorse RFA as primary treatment for large toxic goiters, a combined approach may be an innovative safe solution for reducing the dose of RAI and rapidly controlling local symptoms of hyperthyroidism and compression without undergoing surgical removal of the gland (62). In a pilot study by Chianelli et al., combined LA therapy with RAI treatment induced faster and greater improvement of local and systemic symptoms compared to RAI alone (63). Korkusuz et al. found similar success with combined therapy using MA with RAI for Graves’ disease and large toxic nodular goiters (64). A significant reduction in size was appreciated with restoration of euthyroidism and a reduced dose of RAI (64). Even though MA and LA have proven effective, RFA is more widely disseminated and is better studied to date (62).

Thermoablative procedures can be useful in older patients for providing relief at a rate that may be faster than RAI alone. There are limitations for each treatment modality, however when RAI and RFA are combined, the volume of the nodule is reduced more rapidly. Mader et al., used combined therapy (RAI and RFA) for large goiters which led to a significant reduction in thyroid volume (p<0.05) compared to the control group (RAI mono therapy) (62). However, by three months post treatment the volume reduction did not differ between the two groups (p > 0.05). All patients became euthyroid after treatment and no complications or discomfort were noted (62). With future work, combined therapy may limit the dose of RAI and provide fast relief in patients who refuse or have contraindications to surgery.

## Malignant Thyroid Nodules

Recent studies have demonstrated the efficacy and safety of thermal ablation for low-risk papillary thyroid microcarcinoma (PTMC) (65–76) as well as for recurrent thyroid cancer where the risks of surgery outweigh the benefits or in patients who refuse repeat surgery (77–90). However, it should also be recognized that RFA for low-risk PTMC must be considered in the appropriate context, as many studies demonstrate excellent outcomes and minimal growth with simple active surveillance in this patient population. Preliminary work has not shown benefit for poorly differentiated aggressive tumors such as anaplastic carcinoma (69). For medullary thyroid cancer (MTC), surgery remains the treatment of choice. Few case reports have demonstrated RFA to be a safe and effective option for early MTC in patient’s ineligible for surgery (91) or for patients with a regional recurrence after surgical resection of their MTC (92). However, the data is somewhat limited for MTC and remains controversial.

Careful evaluation of the desired nodule is required before ablation to ensure a successful outcome for the patient and to avoid delay for possible surgery. The main indications include: a) cytopathology confirmed papillary thyroid carcinoma (PTC) without evidence of aggressiveness b) single PTC without extrathyroidal extension c) no metastatic tumors at the time of treatment and d) ineligibility for surgery (78). The operator should note key features during evaluation, such as capsule invasion or lymph node metastases, and whether an aggressive variant of PTC is present. These features should prompt surgery instead of RFA. Currently, the Italian society does not recommend RFA for first line treatment of primary thyroid cancer, however emerging evidence has shown benefits, safety and efficacy for treatment of low-risk tumors (46).

## RFA for Papillary Microcarcinoma (T1aN0M0)

Detection of smaller thyroid cancers, namely PTMCs, is increasing in part because of increased medical imaging and accounts for 50% of papillary thyroid cancers (93). The World Health Organization (WHO) defines PTMC as a small papillary thyroid cancer or < 10 mm in greatest dimension (93). The American Joint Committee on Cancer (AJCC) TNM system classified T1 category (T1: ≤ 2 cm)into T1a: ≤ 1 cm and T1b: ≤ 2 cm (94). Although the 2015 American Thyroid Association guidelines (ATA) do not recommend biopsy of nodules less than 1 cm unless high risk features are present, these small indolent cancers are often incidentally detected (59). On one hand, while surgery is an option, it is an invasive approach for removal of such small indolent lesions that likely would otherwise not have caused significant morbidity. On the other end of the treatment spectrum, several landmark trials have shown no increase in mortality of patients undergoing active surveillance (AS) for small papillary thyroid cancers compared to those who underwent immediate surgery with 10 years of follow up (95, 96). Thus, the optimal management for PTMC is controversial. Some patients may be reluctant to undergo AS with a proven diagnosis of cancer, regardless of its indolence. The unnecessary anxiety of having a cancer diagnosis may make some patients feel more comfortable actively treating their cancer without surgery or observation. Mounting evidence has established short term safety and efficacy of thermal ablation for PTMC (65–73). In addition to surgery and AS, thermal ablative techniques, such as RFA may bridge the gap in treatment options for patients wishing to have their PTMC managed in a minimally invasive way.

Several trials have demonstrated promising results for treating primary PTMC with RFA. Ding et al. used RFA to treat 38 PTMC in 37 patients with a low power setting of 20 W (65). All treated nodules achieved complete ablation, no complications occurred, and no hypothyroidism was encountered. After 12 months post treatment 37 of the 38 nodules were completely resolved with no evidence of nodule recurrence in 37 patients (65). Similarly, Zhang et al. demonstrated safety and efficacy in RFA-treated PTMCs over a 18-month follow-up (66). After treating the lesion(s) with 3-5W of power, a significant volume reduction rate was noted within the first 6 months follow up (p<0.01) but not after the 12 months follow up (66). Of the 98 nodules (92 patients), 10 resolved after six months, and 23 resolved in 12 months. All patients post RFA demonstrated no evidence of residual tumor on ultrasound or histological pathology after US guided biopsy (66). Again, no major complications were noted.

In a meta-analysis examining the efficacy and safety of all ablation techniques for PTMC, RFA showed the highest mean volume reduction rate (99.3%) compared to other thermal ablation techniques such as MA (95.3%) and LA (88.6%) (p<0.001) (75). Although significant heterogeneity between studies is noted, the pooled proportions of complete disappearance of PTMC was 57.6% (95% CI:35.4-79.8) and recurrence was 0.4% (95% CI:0-1.1) (75). Furthermore, the pooled proportions of overall and major complications for all thermal approaches were 3.2% (95% CI:1.1-5.2) and 0.7% (95% CI:0-1.5) demonstrating the safety of these techniques for PTMC (75).

A recent meta-analysis of 12 studies on the efficacy and safety of thermal ablation by RFA, MA or LA included 1,187 patients with 1,284 papillary microcarcinomas. All modalities induced reduction in nodule volume. MW achieved the highest standard mean deviation (-3.82; 95% CI -7.02; - 0.63) than RFA (-1.35; 95% CI -1.62; -1.09) and LA (-1.80; 95% CI -2.75; -0.85), but the difference was not statistically significant. Complete disappearance pooled proportion was 76.2%, 62.9% and 57.3% after RFA, MW and LA treatments, respectively. There was a lower proportion of recurrence after RFA (0.01%) than after MA (0.85%) and LA (1.87%). However, the differences were not statistically significant. The rates of complications observed were also low and similar between the compared techniques (97).

Zhang et al. compared RFA with surgery for patients with a low-risk PTMC, and followed them for five years (67). After 5-year follow-up, RFA was not found to be inferior to surgery with respect to oncological efficacy. Only one patient in the RFA group developed a new lesion (1 of 94) (1.1%) arising in the remaining ipsilateral lobe and none of the RFA group developed lymph node metastases (67). When compared with RFA, surgery took longer, had a longer hospitalization time, and was more costly (p<0.001). The surgical group had a lower thyroid-related quality of life, as well as more complications (2.5% recurrent laryngeal nerve palsy, 1.3% hypoparathyroidism) compared to neither of these complications in the RFA group (p=0.095) (67).

Yan et al. examined the long-term oncological efficacy of RFA in 414 PTMC patients after 42.15 ± 11.88 (range 24 – 69 months) months (76). After RFA, 366 out of 414 tumors (88.41%) completely disappeared, with a volume reduction rate of 98.81 ± 6.41% demonstrating long-term efficacy in this large cohort (76). The incidence of lymph node metastases post RFA was 0.97% (ipsilateral neck in three patients, and one in the central compartment), all of which underwent additional RFA with complete disappearance of the node during follow-up. Recurrent PTMC was found in 10 patients (2.42%); seven were in the contralateral lobe and three in the ipsilateral lobe, which were successfully treated with a second RFA procedure. The mean time of recurrent PTMC development was 27.60 ± 12.71 months (range, 6 – 48 months) (76). Similar to other trials, Yan et al. found no delayed or immediate complications.

As the field of minimally invasive techniques continue to grow, more long-term data will emerge and may substantiate the oncologic effectiveness of thermal ablation for primary microcarcinoma.

## RFA for Papillary Thyroid Cancer (T1bN0M0)

Little evidence exists for thermal ablation of T1bN0M0 cancers, particularly with RFA, compared with T1aN0M0 PTC. The distinction between T1a and T1b is minimal, and the prognosis does not differ significantly between these two subdivisions as demonstrated by previous studies (98, 99). Xiao et al. compared surgery and RFA in patients with T1bN0M0 PTC (91 patients in each group) and found no significant difference between the two groups in local tumor progression and complications (74). In the RFA group, four patients had local tumor progression (4.4%, three persistent PTC and one develop lymph node metastases). Whereas in the surgery group, two patients (2.2%) developed lymph node metastases, and no new or persistent PTC was noted. When the complication rates were compared, the surgery group had four patients with permanent hypoparathyroidism (4.4%) after total thyroidectomy, meanwhile the RFA group developed no major complications, and only two patients experienced moderate pain (74). Even though the mean follow-up time was 25 months, the results are promising. In future studies comparison should be made between RFA treated patients and unilateral thyroid surgery as opposed to total thyroidectomy.

## RFA for Follicular Thyroid Neoplasm

RFA treatment for follicular neoplasm is more controversial than PTC since surgical resection is required to exclude the presence of vascular or capsular invasion to definitively diagnose whether the nodules is a follicular adenoma compared with a carcinoma. However, a recent 5-year study including 10 patients with follicular neoplasm < 2 cm in size found on thyroid biopsy reported that RFA is safe and effective in the short-term for such cases. Ha et al. demonstrate a significant reduction in the mean volume (99.5 ± 1.0%) of lesions, with eight ablated lesions (8/10, 80%) disappearing completely after single treatment on follow-up and no recurrences noted (range: 60-76 months) (100). Further large-scale trials are required to confirm effective oncological control, as it is unclear if sonographically invisible carcinomas still have metastatic potential.

## Recurrent Thyroid Cancer

While the mortality rate for well-differentiated thyroid cancer tends to be favorable, the risk of recurrent PTC can range from 20 – 59% depending on patient and tumor risk factors (78). The mainstay of recurrent thyroid cancer is surgery, followed by RAI therapy and suppressive thyroid hormone replacement treatment, external beam radiotherapy and/or chemotherapy (59). Revision surgery for recurrent disease is not without its challenges due to scarring in the tissue bed, which can make identification and preservation of the RLN and parathyroid glands difficult. Various thermal ablative techniques (RFA, LA or MA) and chemical ablative approaches (ethanol ablation) can be used as an alternative method in patients deemed to be at high surgical risk, those in whom the surgeon deems non-operable or for those refusing to undergo repeat surgery. In each situation, a tailored and multi-disciplinary approach is imperative, as recurrent carcinomas can present in myriad presentations.

In patients treated with lobectomy, total thyroidectomy, and/or neck dissection as their primary surgery, Choi et al. examined patients with local recurrences and compared the efficacy and complication rate between the RFA group and repeat surgery group (77). After propensity score adjustment, the recurrence-free survival rates were comparable between the two groups (p=0.2), including the decrease in the mean serum thyroglobulin levels post treatment (RFA p=0.891 and surgery p=0.963) (77). Additionally, overall complications were noted more frequently in the surgery group, compared with the RFA group, particularly for hypocalcemia (surgery n=27; RFA, n=7;p<0.001) (77), which was similar to the findings of Kim et al. (78).

Various studies have looked at possible parameters to measure the efficacy after RFA for recurrent PTC. Most of these studies have looked at the short-term results (over 6 months), leaving the long-term outcomes still to be determined. Jeong et al. reviewed the literature (79) and found the mean reduction in tumor volume ranging from 50.9% to 8.4% (80–82); with complete disappearance noted in 25-94% of cancers (80, 81, 83–85); therapeutic success rates in 75% - 97% (80, 83, 86, 87); with symptom improvement observed in 64% of patients (82); and a decrease in serum thyroglobulin concentration in most patients signifying therapeutic success (80, 82–84). One study on seventy-three patients reported the mean volume reduction rate of recurrent tumors to be 98.4% ± 6.2%, and the complete disappearance rate 86.1% (78). Furthermore, two meta-analyses including one hundred and eighty-nine patients (87) and two hundred and seventy patients (88) with a local recurrence of their thyroid cancer found a significant decrease in volume and largest diameter of the tumor as well as thyroglobulin level before and after RFA treatment (87, 88).

Longer term data are beginning to emerge, with promising results for treating locally recurrent PTC with US-guided RFA. Chung et al. found after a mean follow up of 80 ± 17.3 months (range, 60-114 months), in a series of twenty-nine patients (forty-six recurrent PTC), that the tumor volume decreased significantly (p <0.001) with a mean volume reduction of 99.5% ± 2.9% and significant thyroglobulin decrease (p<0.001), and complete tumor disappearance of 91.3% by the final evaluation with no delayed complications noted (89).

When recurrences in the central compartment occur, invasion of surrounding structures such as the trachea, esophagus, RLN, and parathyroid glands are more likely, making reoperation in a previously operated tissue bed with a recurrence very challenging. Chung et al. reviewed a series of patients with RFA-treated recurrent thyroid cancer in the central compartment after total thyroidectomy. The mean volume reduction rate was 81.2% ± 55.7%, and complete disappearance of the tumor 72% after a mean follow-up of 47.0 ± 35.4 months (90). The lower rate of tumor disappearance for RFA-treated recurrences in the central compartment can likely be explained by the narrow working space and important neighboring structures. Treatment efficacy was significantly higher for tumors that were not in contact with the trachea, and unsurprisingly, lowest for tumors invading the trachea (90). Thus, the long-term efficacy of RFA treated recurrent papillary thyroid cancer is promising in the right clinical situation. As we anticipate future guidelines in North America, RFA treatment may be indicated for early localized disease to prevent progression into the neighboring vital structures in patients who are poor operative candidates or have other reasons to avoid surgery.

## Complications of RFA

Though multiple studies have demonstrated that thermal ablation is a safe technique, there are some associated complications which are mostly minor. In a meta-analysis of 12 studies including RFA treated benign nodules with follow up for more than three years, Cho et al. found an overall complication rate of 4.6% and a major complication rate of 1.3% in the RFA group (34). In a systematic review including 2786 nodules (24 studies including both benign and recurrent thyroid cancers) the overall RFA complication rate was 2.38%, with 1.35% for major complications (35). On subgroup analysis, the overall and major complication rate was higher for recurrent malignant nodules (10.98%) compared with benign nodules (2.11%) (35).

The most common minor complication include pain with an incidence that ranges from 2.6% to 17.5% in a systematic review that included 32 studies with 3409 patients (101). In a systematic review by Chung et al. transient voice change was one of the common minor complications post-RFA, with an incidence of 0.94% (21/2245 for benign nodules) compared with 7.95%, 14/176 for recurrent cancer (35). Skin burns, hematoma and transient thyroiditis can occur but less frequently (34, 35, 102) (**Table 1**).

**Table 1:** Complications following RFA of benign thyroid nodules [from a systematic review of 3409 patients by Wang et al. (101)].

Complications	N° cases
Pain and sensation of heat	281
Voice changes	32
Hematoma/hemorrhage	31
Vasovagal reactions	19
Nodule rupture	14
Horner syndrome	14
Increase in blood pressure	12
Nausea/vomiting	11
Fever	11
Cough	10
Skin burn	6
Recurrent nerve injury	4
Hypothyroidism	3
Needle track seeding	2
Thyroiditis and thyrotoxicosis	1
Brachial plexus injury	1
Pseudocystic transformation	1

Major complications such as permanent recurrent laryngeal nerve injury, nodule rupture, hematoma requiring surgical drainage, Horner’s syndrome or injury to the adjacent esophagus or trachea are rare (39, 103).

## Conclusion

Given the accumulating international evidence, we anticipate that RFA will one day become routinely used for many benign and malignant conditions of the thyroid. We still require large prospective trials to be performed in diverse populations and with longer term data to substantiate oncologic efficacy. For the novice operator, comfort and ease results from a solid working knowledge of ultrasound guided FNA of the thyroid. Once this has been established, smaller benign nodules may be targeted first before treatment of larger or malignant nodules. Until more robust efficacy data is available for malignant thyroid nodules, RFA for malignant thyroid nodules should ideally be performed in a setting of prospective data analysis and/or clinical trials.

## AUTHOR’S NOTE

This paper was written by members and invitees of the International Head and Neck Scientific Group (www.IHNSG.com).

## Author Contributions

Equal contributions were made by RT and PP-A for the writing of the initial manuscript. JR contributed to the manuscript edits and final review. Members and invitees of the International Head and Neck Scientific Group (www.IHNSG.com) made contributions to the writing and editing of subsequent drafts of the manuscript. All authors contributed to the article and approved the submitted version.

## Conflict of Interest

RT is a consultant for RGS Healthcare.

The authors declare that the research was conducted in the absence of any commercial or financial relationships that could be construed as a potential conflict of interest.

## References

[1] Trimboli P Pelloni F Bini F Marinozzi F Giovanella L. High-Intensity Focused Ultrasound (HIFU) for Benign Thyroid Nodules: 2-Year Follow-Up Results. Endocrine (2019) 65(2):312–7. 10.1007/s12020-019-01909-w 30919288

[2] Guo DM Chen Z Zhai YX Su HH. Comparison of Radiofrequency Ablation and Microwave Ablation for Benign Thyroid Nodules; A Systematic Review and Meta-Analysis. Clin Endocrinol (Oxf) (2021) 8 187–96. 10.1111/cen.14438 33556187

[3] Guglielmi R Pacella CM Bianchini A Bizzarri G Rinaldi R Graziano FM. Percutaneous Ethanol Injection Treatment in Benign Thyroid Lesions: Role and Efficacy. Thyroid (2004) 14(2):125–31. 10.1089/105072504322880364 15068627

[4] Sung JY Kim YS Choi H Lee JH Baek JH. Optimum First-Line Treatment Technique for Benign Cystic Thyroid Nodules: Ethanol Ablation or Radiofrequency Ablation? AJR Am J Roentgenol (2011) 196(2):W210–4. 10.2214/AJR.10.5172 21257865

[5] Kim JH Lee HK Lee JH Ahn IM Choi CG. Efficacy of Sonographically Guided Percutaneous Ethanol Injection for Treatment of Thyroid Cysts Versus Solid Thyroid Nodules. AJR Am J Roentgenol (2003) 180(6):1723–6. 10.2214/ajr.180.6.1801723 12760950

[6] Lim HK Lee JH Ha EJ Sung JY Kim HK Baek JH. Radiofrequency Ablation of Benign non-Functioning Thyroid Nodules: 4 Year Follow-Up Results for 111 Patients. Eur Radiol (2013) 23(4):1044–9. 10.1007/s00330-012-2671-3 23096937

[7] Bernardi S Giudici F Cesareo R Antonelli G Cavallaro M Deandrea M. Five-Year Results of Radiofrequency and Laser Ablation of Benign Thyroid Nodules: A Multicenter Study From the Italian Minimally Invasive Treatments of the Thyroid Group. 2020. Thyroid (2020) 30:1759–70. 10.1089/thy.2020.0202 32578498

[8] Bernardi S Dobrinja C Fabris B Bazzocchi G Sabato N Ulcigrai V. Radiofrequency Ablation Compared to Surgery for the Treatment of Benign Thyroid Nodules. Int J Endocrinol (2014) 934595:1–10. 10.1155/2014/934595 PMC4090443 25045352

[9] Che Y Jin S Shi C Wang L Zhang X Li Y. Treatment of Benign Thyroid Nodules: Comparison of Surgery With Radiofrequency Ablation. AJNR Am J Neuroradiol (2015) 36:1321–5. 10.3174/ajnr.A4276 PMC7965284 25814656

[10] Cesareo R Naciu AM Iozzino M Pasqualini V Simeoni C Casini A. Nodule Size as Predictive Factor of Efficacy of Radiofrequency Ablation in Treating Autonomously Functioning Thyroid Nodules. Int J Hyperthermia (2018) 34:617–23. 10.1080/02656736.2018.1430868 29357717

[11] Cappelli C Franco F Pirola I Gandossi E Marini F Di Lodovico E. Radiofrequency Ablation of Functioning and non-Functioning Thyroid Nodules: A Single Institution 12-Month Survey. J Endocrinol Invest (2020) 43:477–82. 10.1007/s40618-019-01132-4 31654311

[12] Cesareo R Palermo A Benvenuto D Cella E Pasqualini V Bernardi S. Efficacy of Radiofrequency Ablation in Autonomous Functioning Thyroid Nodules. A Systematic Review and Meta-Analysis. Rev Endocrine Metab Disord (2019) 20(1):37–44. 10.1007/s11154-019-09487-y 30887407

[13] Sung JY Baek JH Jung SL Kim JH Kim KS Lee D. Radiofrequency Ablation for Autonomously Functioning Thyroid Nodules: A Multicenter Study. Thyroid (2015) 25:112–. 10.1089/thy.2014.0100 25320840

[14] Baek JH Moon WJ Kim YS Lee JH Lee D. Radiofrequency Ablation for the Treatment of Autonomously Functioning Thyroid Nodules. World J Surg (2009) 33:1971–7. 10.1007/s00268-009-0130-3 19575141

[15] Bernardi S Stacul F Michelli A Giudici F Zuolo G de Manzini N. 12-Month Efficacy of a Single Radiofrequency Ablation on Autonomously Functioning Thyroid Nodules. Endocrine (2017) t 57(3):402–8. 10.1007/s12020-016-1174-4 27848197

[16] Lee GM You JY Kim HY Chai YJ Kim HK Dionigi G. Successful Radiofrequency Ablation Strategies for Benign Thyroid Nodules. Endocrine (2019) 64(2):316–21. 10.1007/s12020-018-1829-4 30569260

[17] Kim JH Baek JH Lim HK Ahn HS Baek SM Choic YJ. 2017 Thyroid Radiofrequency Ablation Guideline: Korean Society of Thyroid Radiology. Thyroid (2018) 19(4):632–55. 10.3348/kjr.2018.19.4.632 PMC6005940 29962870

[18] Huh JY Baek JH Choi H Kim JK Lee JH. Symptomatic Benign Thyroid Nodules: Efficacy of Additional Radiofrequency Ablation Treatment Sessions – Prospective Randomized Study. Radiology (2012) 263(3):909–16. 10.1148/radiol.12111300 22438360

[19] Fuller CW Nguyen SA Lohia S Gillespie MB. 2014 Radiofrequency Ablation for Treatment of Benign Thyroid Nodules: Systematic Review. Laryngoscope (2014) 124(1):346–53. 10.1002/lary.24406 24122763

[20] Jeong WK Baek JH Rhim H Kim YS Kwak MS Jeong HJ. Radiofrequency Ablation of Benign Thyroid Nodules: Safety and Imaging Follow-Up in 236 Patients. Eur Radiol (2008) 18(6):1244–50. 10.1007/s00330-008-0880-6 18286289

[21] Jung SL Baek JH Lee JH Shong YK Sung JY Kim KS. Efficacy and Safety of Radiofrequency Ablation for Benign Thyroid Nodules: A Prospective Multicenter Study. Korean J Radiol (2018) 19(1):167–74. 10.3348/kjr.2018.19.1.167 PMC5768499 29354014

[22] Dobnig H Zechmann W Hermann M Lehner M Heute D Mirzaei S. Radiofrequency Ablation of Thyroid Nodules: “Good Clinical Practice Recommendations” for Austria: An Interdisciplinary Statement From the Following Professional Associations: Austrian Thyroid Association (Osdg), Austrian Society for Nuclear Medicine and Molecular Imaging (Ognmb), Austrian Society for Endocrinology and Metabolism (Oges), Surgical Endocrinology Working Group (ACE) of the Austrian Surgical Society (Oegch). Wien Med Wochenschr (2020) 170(102):6–14. 10.1007/s10354-019-0682-2 30725443

[23] Gharib H Hegedus L Pacella CM Baek JH Papini E. Nonsurgical, Image-Guided, Minimally Invasive Therapy for Thyroid Nodules. J Clin Endocrinol Metab (2013) 98(10):3949–57. 10.1210/jc.2013-1806 23956350

[24] Ha EJ Baek JH Kim KW Pyo J Lee JH Baek SH. Comparative Efficacy of Radiofrequency and Laser Ablation for the Treatment of Benign Thyroid Nodules: Systematic Review Including Traditional Pooling and Bayesian Network Meta-Analysis. J Clin Endocrinol Metab (2015) 100(5):1903–11. 10.1210/jc.2014-4077 25695887

[25] Mauri G Cova L Monaco CG Sconfienza LM Corbetta S Benedini S. Benign Thyroid Nodules Treatment Using Percutaneous Laser Ablation (PLA) and Radiofrequency Ablation (RFA). Int J Hyperthermia (2017) 33(3):295–9. 10.1080/02656736.2016.1244707 27701923

[26] Chen F Tian G Kong D Zhong L Jian T. Radiofrequency Ablation for Treatment of Benign Thyroid Nodules. A PRISMA-Compliant Systematic Review and Meta-Analysis of Outcomes. Med (Baltimore) (2016) 95(34):e4659. 10.1097/MD.0000000000004659 PMC5400335 27559968

[27] Gharib H Papini E Garber JR Garber JR Duick DS Harrell RM. Aace/Ace/Ame Task Force on Thyroid Nodules. American College of Endocrinology, and Associazione Medici Endocrinologi Medical Guidelines for Clinical Practice for the Diagnosis and Management of Thyroid Nodules - 2016 Update. Endocr Pract (2016) 22(5):622–39. 10.4158/EP161208.GL 27167915

[28] Deandrea M Sung JY Limone P Mormile A Garino F Ragazzoni F. Efficacy and Safety of Radiofrequency Ablation Versus Observation for Nonfunctioning Benign Thyroid Nodules: A Randomized Controlled International Collaborative Trial. Thyroid (2015) 25(8):890–6. 10.1089/thy.2015.0133 26061686

[29] Cesareo R Pasqualini V Simeoni C Sacchi M Saralli E Campagna G. Prospective Study of Effectiveness of Ultrasound-Guided Radiofrequency Ablation Versus Control Group in Patients Affected by Benign Thyroid Nodules. J Clin Endocrinol Metab (2015) 100(2):460–6. 10.1210/jc.2014-2186 25387256

[30] Trimboli P Castellana M Sconfienza LM Virilli C Pescatori LC Cesareo R. Efficacy of Thermal Ablation in Benign non-Functioning Solid Thyroid Nodule: A Systematic Review and Meta-Analysis. Endocrine (2020) Jan 67(1):35–43. 10.1007/s12020-019-02019-3 31327158

[31] Deandrea M Trimboli P Garino F Mormile A Magliona G Rumunni MJ. Long-Term Efficacy of a Single Session of RFA for Benign Thyroid Nodules: A Longitudinal 5-Year Observational Study. J Clin Endocrinol Metab (2019) Sept 104(9):3751–6. 10.1210/jc.2018-02808 30860579

[32] Deandrea M Limone P Basso E Mormile A Ragazzoni F Gamarra E. US-Guided Percutaneous Radiofrequency Thermal Ablation for the Treatment of Solid Benign Hyperfunctioning or Compressive Thyroid Nodules. Ultrasound Med Biol (2008) 34(5):784–91. 10.1016/j.ultrasmedbio.2007.10.018 18207307

[33] Faggiano A Ramundo V Assanti AP Fonderico F Macchia PE Misso C. Thyroid Nodules Treated With Percutaneous Radiofrequency Thermal Ablation: A Comparative Study. J Clin Endocrinol Metab (2012) 97(12):4439–45. 10.1210/jc.2012-2251 23019349

[34] Chung SR Suh CH Baek JH Park HS Choi YJ Lee JH. Safety of Radiofrequency Ablation of Benign Thyroid Nodules and Recurrent Thyroid Cancers: A Systematic Review and Meta-Analysis. Int J Hyperthermia (2017) 33(8):920–30. 10.1080/02656736.2017.1337936 28565997

[35] Cho SJ Baek JH Chung SR Young JC Lee JH. Long Term Results of Thermal Ablation of Benign Thyroid Nodules: A Systematic Review and Meta-Analysis. Endocrinol Metab (2020) 35(2):339–50. 10.3803/EnM.2020.35.2.339 PMC7386110 32615718

[36] Papini E Monpeyssen H Frasoldati A Hegedus L. European Thyroid Association Clinical Guideline for the Use of Image-Guided Ablation in Benign Thyroid Nodules. Eur Thyroid J (2020) 9(4):172–85. 10.1159/000508484 PMC7445670 32903999

[37] Baek JH Ha EJ Choi YJ Sung JY Kim JK Shong YK. Radiofrequency Versus Ethanol Ablation for Treating Predominantly Cystic Thyroid Nodules: A Randomized Clinical Trial. Korean J Radiol (2015) 16(6):1332–40. 10.3348/kjr.2015.16.6.1332 PMC4644756 26576124

[38] Papini E Pacella CM Solbiati LA Achille G Barbaro D Bernardi S. Minimally-Invasive Treatments for Benign Thyroid Nodules: A Delphi-based Consensus Statement From the Italian Minimally-Invasive Treatments of the Thyroid (MITT) Group. Int J Hyperthermia (2019) 36(1):376–82. 10.1080/02656736.2019.1575482 30909759

[39] Muhammad H Santhanam P Russell JO Kuo JH. RFA and Benign Thyroid Nodules: Review of the Current Literature. Laryngoscope Investig Otolaryngol (2021) 6(1):155–65. 10.1002/lio2.517 PMC7883624 33614945

[40] Hall WH McGahan JP Link DP deVere White RW. Combined Embolization and Percutaneous Radiofrequency Ablation of a Solid Renal Tumor. Am J Roentgenol (2000) 174(6):1592–4. 10.2214/ajr.174.6.1741592 10845488

[41] Steinke K. Radiofrequency Ablation of Pulmonary Tumours: Current Status. Cancer Imaging (2008) 8(1):27–35. 10.1102/1470-7330.2008.0008 18331970 PMC2267693

[42] McGahan JP Browning PD Brock JM Tesluk H. Hepatic Ablation Using Radiofrequency Electrocautery. Invest Radiol (1990) 25(3):267–70. 10.1097/00004424-199003000-00011 2185179

[43] Rosenthal DJ Hornicek FJ Wolfe MW Jennings LC Gebhardt MC Mankin HJ. Percutaneous Radiofrequency Coagulation of Osteoid Osteoma Compared With Operative Treatment. J Bone Joint Surg (1998) 80(6):815–21. 10.2106/00004623-199806000-00005 9655099

[44] Sousa J El-Atassi R Rosenheck S Calkins Langberg J Morady F. Radiofrequency Catheter Ablation of the Atrioventricular Junction From the Left Ventricle. Circulation (1991) 84(2):567–71. 10.1161/01.cir.84.2.567 1860201

[45] Ha EJ Baek JH Lee JH. Moving-Shot Versus Fixed Electrode Techniques for Radiofrequency Ablation: Comparison in an Ex-Vivo Bovine Liver Tissue Model. Korean J Radiol (2014) 15(6):836–43. 10.3348/kjr.2014.15.6.836 PMC4248641 25469097

[46] Garberoglio R Aliberti C Appetecchia M Attard M Boccuzzi G Boraso F. Radiofrequency Ablation for Thyroid Nodules: Which Indications? first Ital Opin statement J Ultrasound (2015) 18:423–30. 10.1007/s40477-015-0169-y PMC4630279 26550079

[47] National Institute for Health and Care Excellence. Ultrasound Guided Percutaneous Radiogrequency Ablation for Benign Thyroid Nodules. In: . Interventional Procedure Guidance [IPT562]. London: National Institute for Health and Care Excellence (2016). Available at: https://www.nice.org.uk/guidance/ipg562.

[48] Mauri G Pacella CM Papini E Solbiati L Goldberg SN Ahmed M. Image-Guided Thyroid Ablation: Proposal for Standardization of Terminology and Reporting Criteria. Thyroid (2019) May 29(5):611–8. 10.1089/thy.2018.0604 30803397

[49] Park HS Baek JH Park AW Chung SR Choi YJ Lee JH. Thyroid Radiofrequency Ablation: Updates on Innovative Devices and Techniques. Korean J Radiol (2017) 18(4):615–23. 10.3348/kjr.2017.18.4.615 PMC5447637 28670156

[50] Deandra M Garino F Alberto M Garberoglio R Rossetto R Bonelli N. Radiofrequency Ablation for Benign Thyroid Nodules According to Different Ultrasound Features: An Italian Multicenter Prospective. Eur J Endocrinol (2019) 180:79–87. 10.1530/EJE-18-0685 30407921

[51] Spiezia S Garberoglio R Milone F Ramundo V Caiazzo C Assanti AP. Thyroid Nodules and Related Symptoms are Stably Controlled Two Years After Radiofrequency Thermal Ablation. Thyroid (2009) Mar 19(3):219–25. 10.1089/thy.2008.0202 19265492

[52] Baek JH Kim YS Lee D Huh JY Lee JH. Benign Predominantly Solid Thyroid Nodules: Prospective Study of Efficacy of Sonographically Guided Radiofrequency Ablation Versus Control Conditions. AJR Am J Roentgenol (2010) Apr 194(4):1137–42. 10.2214/AJR.09.3372 20308523

[53] Sim JS Baek JH Lee J Cho W Jung SI. Radiofrequency Ablation of Benign Thyroid Nodules: Depicting Early Sign of Regrowth by Calculating Vital Volume. Int J Hyperthermia (2017) 33(8):905–10. 10.1080/02656736.2017.1309083 28540795

[54] Trimboli P Deandrea M. Treating Thyroid Nodules by Radiofrequency: Is the Delivered Energy Correlated With the Volume Reduction Rate? A pilot study Endocrine (2020) 69(3):682–7. 10.1007/s12020-020-02275-8 32319012

[55] Cervelli R Mazzeo S Boni G Boccuzzi A Bianchi F Brozzi F. Comparison Between Radioiodine Therapy and Single-Session Radiofrequency Ablation of Autonomously Functioning Thyroid Nodules: A Retrospective Study. Clin Endocrinol (2019) 90:608–16. 10.1111/cen.13938 30657603

[56] Kim HJ Cho SJ Baek JH Suh CH. Efficacy and Safety of Thermal Ablation for Autonomously Functioning Thyroid Nodules: A Systematic Review and Meta-Analysis. Eur Radiol (2021) 31(2):605–15. 10.1007/s00330-020-07166-0 32816198

[57] Guan SH Wang H Teng DK. Comparison of Ultrasound-Guided Thermal Ablation and Conventional Thyroidectomy for Benign Thyroid Nodules: A Systematic Review and Meta-Analysis. Int J Hyperthermia (2020) 37:442–9. 10.1080/02656736.2020.1758802 32369708

[58] Jin H Lin W Lu L Cui M. Conventional Thyroidectomy vs Thyroid Thermal Ablation on Postoperative Quality of Life and Satisfaction for Patients With Benign Thyroid Nodules. Eur J Endocrinol (2021) 184(1):131–41. 10.1530/EJE-20-0562 33112273

[59] Haugen BR Alexander EK Bible KC Doherty GM Mandel SJ Nikiforov YE. American Thyroid Association Management Guidelines for Adult Patients With Thyroid Nodules and Differentiated Thyroid Cancer: The American Thyroid Association Guidelines Task Force on Thyroid Nodules and Differentiated Thyroid Cancer. Thyroid (2016) 26:1–133. 10.1089/thy.2015.0020 26462967 PMC4739132

[60] Chandrasekhar S Randolph GW Seidman MS Rosenfeld R Angelos P Barkmeier-Kraemer JS. American Academy of Otolaryngology Head and Neck Surgery Clinical Practice Guideline: Improving Voice Outcomes After Thyroid Surgery. Otolaryngol Head Neck Surg (2013) 148(6 Suppl):S1–37. 10.1177/0194599813487301 23733893

[61] Bo XW Lu F Xu HX Sun LP Zhang K. Thermal Ablation of Benign Thyroid Nodules and Papillary Thyroid Microcarcinoma. Front Oncol (2020) 10:580431. 10.3389/fonc.2020.580431 33194708 PMC7658440

[62] Mader A Mader OM Groner D Korkusuz Y Ahmad S Grunwald F. Minimally Invasive Local Ablative Therapies in Combination With Radioiodine Therapy in Benign Thyroid Disease: Preparation, Feasibility and Efficiency – Preliminary Results. Int J Hyperthermia (2017) 33(8):895–904. 10.1080/02656736.2017.1320813 28540810

[63] Chianelli M Bizzarri V Todino I Misischi A Bianchini F Graziano R. Laser Ablation and 131-Iodine: A 24-Month Pilot Study of Combined Treatment for Large Toxic Nodular Goiter. J Clin Endocrinol Metab (2014) 99(7):E1283–6. 10.1210/jc.2013-2967 24684455

[64] Korkusuz H Happel C Koch DA Gruenwald F. Combination of Ultrasound-Guided Percutaneous Microwabe Ablation and Radioiodine Therapy in Benign Thyroid Disease: A 3-Month Follow Up Study. RoFo (2016) 188(1):60–8. 10.1055/s-0041-106538 26566268

[65] Ding M Tang X Cui D Chi J Shi Y Wang T. Clinical Outcomes of Ultrasound-Guided Radiofrequency Ablation for the Treatment of Primary Papillary Thyroid Microcarcinoma. Clin Radiol (2019) 74:712–7. 10.1016/j.crad.2019.05.012 31253420

[66] Zhang M Luo Y Zhang Y Tang J. Efficacy and Safety of Ultrasound-Guided Radiofrequency Ablation for Treating Low-Risk Papillary Thyroid Microcarcinoma: A Prospective Study. Thyroid (2016) 26:11:1581–7. 10.1089/thy.2015.0471 27445090

[67] Zhang M Tufano RP Russell JO Zhang Y Zhang Y Qiao Z. Ultrasound-Guided Radiofrequency Ablation Versus Surgery for Low-Risk Papillary Thyroid Microcarcinoma: Results of Over 5 Years’ Follow-Up. Thyroid (2020) 30:3:408–17. 10.1089/thy.2019.0147 31910107

[68] Papini E Guglielmi R Hosseim G Misischi I Graziano F Chianelli M. Ultrasound-Guided Laser Ablation of Incidental Papillary Thyroid Microcarcinoma: A Potential Therapeutic Approach in Patients With at Surgical Risk. Thyroid (2011) 21(8):917–20. 10.1089/thy.2010.0447 PMC3148119 21595556

[69] Jeong SY Baek JH Choi YJ Chung SR Sung TY Kim WG. Radiofrequency Ablation of Primary Thyroid Carcinoma: Efficacy According to the Types of Thyroid Carcinoma. Int J Hyperthermia (2018) 34:611–6. 10.1080/02656736.2018.1427288 29322881

[70] Teng D Sui G Liu C Wang Y Xia Y Wang H. Long-Term Efficacy of Ultrasound-Guided Low Power Microwave Ablation for the Treatment of Primary Papillary Thyroid Microcarcinoma: A 3 Year Follow Up Study. J Cancer Res Clin Oncol (2018) 144(4):771–9. 10.1007/s00432-018-2607-7 PMC11813516 29427209

[71] Yue W Wang S Yu S Wang B. Ultrasound-Guided Percutaneous Microwave Ablation of Solitary T1N0M0 Papillary Thyroid Microcarcinoma: Initial Experience. Int J Hyperthermia (2014) 30:150–7. 10.3109/02656736.2014.885590 24571178

[72] Zhang L Zhou W Zhan W Peng Y Jiang S Xu S. Percutaneous Laser Ablation of Unifocal Papillary Thyroid Microcarcinoma: Utility of Conventional Ultrasound and Contrast-Enhanced Ultrasound in Assessing Local Therapeutic Response. World J Surg (2018) 42:2476–84. 10.1007/s00268-018-4500-6 29488064

[73] Kim JH Baek JH Sung JY Min HS Kim KW Hah JH. Radiofrequency Ablation of Low-Risk Small Papillary Thyroid Carcinoma: Preliminary Results for Patients Ineligible for Surgery. Int J Hyperthermia (2017) 33:212–9. 10.1080/02656736.2016.1230893 27590679

[74] Xiao J Zhang Y Zhang M Lan Y Yan L Luo Y. Ultrasonography-Guided Radiofrequency Ablation vs. Surgery for the Treatment of Solitary T1bN0M0 Papillary Thyroid Carcinoma: A Comparative Study. Clin Endocrinol (Oxf) (2021) 94(4):684–91. 10.1111/cen.14361 33128786

[75] Choi Y Jung SL. Efficacy and Safety of Thermal Ablation Techniques for the Treatment of Primary Papillary Thyroid Microcarcinoma: A Systematic Review and Meta-Analysis. Thyroid (2020) 5:720–31. 10.1089/thy.2019.0707 31801432

[76] Yan L Lan Y Xiao J Lin L Jiang B Luo Y. Long-Term Outcomes of Radiofrequency Ablation for Unifocal Low-Risk Papillary Thyroid Microcarcinoma: A Large Cohort Study of 414 Patients. Eur Radiol (2021) 31(2):685–94. 10.1007/s00330-020-07128-6 32813103

[77] Choi Y Jung SL Bae JS Lee SH Jung CK Jang J. Comparison of Efficacy and Complications Between Radiofrequency Ablation and Repeat Surgery in the Treatment of Locally Recurrent Thyroid Cancers: A Single-Center Propensity Score Matching Study. Int J Hyperthermia (2019) 36:1:358–366. 10.1080/02656736.2019.1571248 30836037

[78] Kim JH Yoo WS Park YJ Park DJ Yun TJ Choi SH. Efficacy and Safety of Radiofrequency Ablation for Treatment of Locally Recurrent Thyroid Cancers Smaller Than 2 Cm. Radiology (2015) 276(3):909–18. 10.1148/radiol.15140079 25848897

[79] Jeong SY Baek JH Choi YJ Lee JH. Ethanol and Thermal Ablation for Malignant Thyroid Tumors. Int J Hyperthermia (2017) 33(8):938–45. 10.1080/02656736.2017.1361048 28797186

[80] Baek JH Kim YS Sung JY Choi H Lee JH. Locoregional Control of Metastatic Well-Differentiated Thyroid Cancer by Ultrasound-Guided Radiofrequency Ablation. AJR Am J Roentgenol (2011) 197(2):W331–6. 10.2214/AJR.10.5345 21785061

[81] Lee SJ Jung SL Kim BS Ahn KJ Choi HS Lim DJ. Radiofrequency Ablation to Treat Loco-Regional Recurrence of Well-Differentiated Thyroid Carcinoma. Korean J Radiol (2014) 15(6):817–26. 10.3348/kjr.2014.15.6.817 PMC4248639 25469095

[82] Park KW Shin JH Han BK Ko EY Chung JH. Inoperable Symptomatic Recurrent Thyroid Cancers: Preliminary Result of Radiofrequency Ablation. Ann Surg Oncol (2011) 18:2564–8. 10.1245/s10434-011-1619-1 21347777

[83] Dupuy DE Monchik JM Decrea C Pisharodi L. Radiofrequency Ablation of Regional Recurrence From Well-Differentiated Thyroid Malignancy. Surgery (2001) Dec 130(6):971–7. 10.1067/msy.2001.118708 11742325

[84] Monchik JM Donatini G Iannuccilli J Dupuy D. Radiofrequency Ablation and Percutaneous Ethanol Injection Treatment for Recurrent Local and Distant Well-Differentiated Thyroid Carcinoma. Ann Surg (2006) 244:296–304. 10.1097/01.sla.0000217685.85467.2d 16858194 PMC1602167

[85] Wang L Ge M Xu D Chen L Qian C Shi K. Ultrasonography-Guided Percutaneous Radiofrequency Ablation for Cervical Lymph Node Metastasis From Thyroid Carcinoma. J Cancer Res Ther (2014) 10 suppl:C144–9. 10.4103/0973-1482.145844 25450273

[86] Lim HK Baek JH Lee JH Kim WB Kim TY Shong YK. Efficacy and Safety of Radiofrequency Ablation for Treating Locoregional Recurrence From Papillary Thyroid Cancer. Eur Radiol (2015) 25(1):163–70. 10.1007/s00330-014-3405-5 25199815

[87] Zhao Q Tian G Kong D Jiang T. Meta-Analysis of Radiofrequency Ablation for Treating the Local Recurrence of Thyroid Cancers. J Endocrinol Invest (2016) 39(8):909–16. 10.1007/s40618-016-0450-8 26980591

[88] Suh CH Baek JH Lee JH Choi YJ Lee JH. Efficacy and Safety of Radiofrequency Ablation and Ethanol Ablation for Treating Locally Recurrent Thyroid Cancer: A Systematic Review and Meta-Analysis. Thyroid (2016) 26(3):420–8. 10.1089/thy.2015.0545 26782174

[89] Chung SR Baek JH Choi YJ Lee JH. Longer-Term Outcomes of Radiofrequency Ablation for Locally Recurrent Papillary Thyroid Cancer. Eur Radiol (2019) Sep 29(9):4897–903. 10.1007/s00330-019-06063-5 30805701

[90] Chung SR Baek JH Choi YJ Sung TY Song DE Kim TY. Efficacy of Radiofrequency Ablation for Recurrent Thyroid Cancer Invading the Airways. Eur Radiol (2021) 31(4):2153–60. 10.1007/s00330-020-07283-w 32945966

[91] Biamonte E Solbiati L Ierace T Colombo P Lavezzi E Mazziotti G. Medullary Thyroid Carcinoma Treated With Percutaneous Ultrasound-Guided Radiofrequency Ablation. Endocrine (2019) 65(3):515–9. 10.1007/s12020-019-01995-w 31273680

[92] Tong MY Li HS Che Y. Recurrent Medullary Thyroid Carcinoma Treated With Percutaneous Ultrasound-Guided Radiofrequency Ablation: A Case Report. World J Clin Cases (2021) 9(4):864–70. 10.12998/wjcc.v9.i4.864 PMC7852637 33585633

[93] Noguchi S Yamashita H Uchino S Watanabe S. Papillary Microcarcinoma. World J Surg (2008) 32(5):747–53. 10.1007/s00268-007-9453-0 PMC2323028 18264828

[94] Tuttle RM Haugen B Perrier ND. Updated American Joint Committee on Cancer/Tumor-Node-Metastasis Staging System for Differentiated and Anaplastic Thyroid Cancer (Eighth Edition): What Changed and Why? Thyroid (2017) 27(6):751–6. 10.1089/thy.2017.0102 PMC5467103 28463585

[95] Ito Y Miyauchi A Inoue H Fukushima M Kihara M Higashiyama T. An Observational Trial for Papillary Thyroid Microcarcinoma in Japanese Patients. World J Surg (2010) 34(1):28–35. 10.1007/s00268-009-0303-0 20020290

[96] Oda H Miyachi A Ito Y Yoshioka K Nakayama A Sasai H. Incidences of Unfavorable Events in the Management of Low-Risk Papillary Microcarcinoma of the Thyroid by Active Surveillance Versus Immediate Surgery. Thyroid (2016) 26(1):150–5. 10.1089/thy.2015.0313 PMC4739129 26426735

[97] Tong M Li S Li Y Li Y Feng Y Che Y. Efficacy and Safety of Radiofrequency, Microwave and Laser Ablation for Treating Papillary Thyroid Microcarcinoma: A Systematic Review and Meta-Analysis. Int J Hyperthermia (2019) 36(1):1278–86. 10.1080/02656736.2019.1700559 31826684

[98] Anderson KL Youngwirth LM Scheri RP Stang MT Roman SA Sosa JA. T1a Versus T1b Differentiated Thyroid Cancers: do We Need to Make the Distinction? Thyroid (2016) 8:1046–52. 10.1089/thy.2016.0073 PMC4976229 27266722

[99] Wang LY Nixon IJ Palmer FL Thomas D Tuttle RM Shaha A. Comparable Outcomes for Patients With pT1a and pT1b Differentiated Thyroid Cancer: Is There a Need for Change in the AJCC Classification System? Surgery (2014) 156(6):1484–90. 10.1016/j.surg.2014.08.037 25456937

[100] Ha SM Sung JY Baek JH Na DG Kim JH Yoo H. Radiofrequency Ablation of Small Follicular Neoplasms: Initial Clinical Outcomes. Int J Hyperthermia (2017) 33(8):931–7. 10.1080/02656736.2017.1331268 28545338

[101] Wang JF Wu T Hu KP Xu W Zheng BW Zheng BW. Complications Following Radiofrequency Ablation of Benign Thyroid Nodules: A Systematic Review. Chin Med J(Engl) (2017) 130(11):1361–70. 10.4103/0366-6999.206347 PMC5455047 28524837

[102] Bernardi S Lanzilotti V Papa G Panizzo N Dobrinja C Fabris B. Full-Thickness Skin Burn Caused by Radiofrequency Ablation of a Benign Thyroid Nodule. Thyroid (2016) 26(1):183–4. 10.1089/thy.2015.0453 PMC4742967 26561213

[103] Ben Hamou A Ghanassia E Espiard S Rached HA Jannin A Correas JM. Safety and Efficacy of Thermal Ablation (Radiofrequency and Laser): Should We Treat All Types of Thyroid Nodules? Int J Hyperthermia (2019) 36(1):666–76. 10.1080/02656736.2019.1627432 31317800

